# MDA5-positive dermatomyositis without muscle weakness and rash: a case report and literature review

**DOI:** 10.3389/fmed.2024.1482350

**Published:** 2024-12-16

**Authors:** Ping-Ping Xiao, Wei Fan, Xu-Yan Chen, Ke-Cong Li, Ting-Ting Li, Zhi-Gao Dong

**Affiliations:** ^1^Department of Hematology and Rheumatology, The Second Affiliated Hospital of Xiamen Medical College, Xiamen, China; ^2^Department of Thoracic Oncology, The Second Affiliated Hospital of Xiamen Medical College, Xiamen, China; ^3^Department of Radiology, The Second Affiliated Hospital of Xiamen Medical College, Xiamen, China

**Keywords:** melanoma differentiation-associated gene 5, dermatomyositis, interstitial lung disease, interferon, case report

## Abstract

Melanoma differentiation-associated gene 5 (MDA5)-positive dermatomyositis (DM) is a rare systemic autoimmune disease associated with a high rate of mortality attributable to rapidly progressive interstitial lung disease. MDA5-positive DM is often complicated by a typical rash and muscle weakness. Herein, we describe a 50-year-old woman who presented with worsening pulmonary symptoms with an absence of typical clinical characteristics. We also review the treatments and prognosis made in similar cases, highlighting that closer attention should be paid to patients presenting with atypical DM, particularly when clinical manifestations such as rash and muscle weakness are not apparent.

## 1 Introduction

Melanoma differentiation-associated gene 5 (MDA5)-positive dermatomyositis (DM) is a rare disease (1). Although most cases tend to be characterized by a typical rash and elevated or normal muscle enzyme levels, patients are frequently misdiagnosed or experience a delayed diagnosis because of an absence of physical symptoms such as a rash and muscle weakness (2). In addition to the detection of specific antibodies against MDA5, many non-specific clinical symptoms lead to misdiagnosis or delayed diagnosis. Non-specific clinical symptoms such as cough, may be misdiagnosed as chronic obstructive pulmonary disease, and fever, which may be misdiagnosed with such ailments as a bacterial, fungal, or viral infection. Accordingly, particular attention should be paid to patients with atypical DM. Herein, we describe a patient who lacked the typical clinical characteristics of MDA5-positive DM and was ultimately diagnosed with the disorder following multidisciplinary consultation. Similar cases reported in recent years are also reviewed.

## 2 Case presentation

A 50-year-old woman presented with expectoration and coughing that had persisted for 2 months, along with wheezing that had occurred in the previous month. She had also experienced intermittent fever ranging from 37.6 to 39.0°C. The patient reported a small amount of white sputum when coughing; however, she denied any symptoms of dyspnea. The patient was a farmer who had never smoked, had a history of chronic gastritis, she had no history or family history of respiratory diseases. Upon physical examination, the patient was found to be febrile with a temperature of 38.0°C, peripheral oxygen saturation of 96% in room air, breathing rate of 20 breaths/min, blood pressure of 115/86 mmHg, and heart rate of 84 beats/min. Lung and chest auscultations revealed wet rales and reduced entry of air into the lung bases, respectively, whereas an electrocardiogram showed sinus rhythm. There was no evidence of constitutional syndrome, dysphagia, finger clubbing, erythema around the fingernail, skin ulcers, synovitis, Raynaud’s syndrome, or muscle weakness. Muscle strength of the limbs was normal, as determined by standardized manual muscle testing.

An initial blood test revealed an elevated white blood cell count of 12.49 × 10^9^/L (normal range [NR]: 3.5–9.5 × 10^9^/L), a neutrophil cell count of 10.14 (NR: 1.8–6.3 × 10^9^/L), and a normal lymphocyte cell count of 1.61 (NR: 1.1–3.2 × 10^9^/L). Procalcitonin and C-reactive protein (CRP) levels were normal. The renal profile was also within the normal range, as was the brain natriuretic peptide level of 16.50 pg./mL (NR: 0–100 pg/mL). Blood culture, urinary *Legionella* test, and pneumococcal antigen test results were all negative. Similarly, the 2019-Novel Coronavirus (2019-nCoV) polymerase chain reaction results were negative on three separate occasions. However, elevated levels of the following serum enzyme were detected: glutamic oxaloacetic transaminase, 62 IU/L (NR: 13.0–35.0 IU/L); glutamic pyruvic transaminase, 93 IU/L (NR: 7.0–40.0 IU/L); *γ*-glutamyl transpeptidase, 147 IU/L (NR: 7.0–45.0 IU/L); and lactate dehydrogenase (LDH), 337 IU/L (NR: 100.0–240.0 IU/L), ferritin elevated in the level of 708.20 ng/mL (NR: 11.0–306.80 IU/L). Creatine kinase and creatine kinase isoenzyme levels were within the normal ranges. The erythrocyte sedimentation rate was 25 mm/h (NR: 0–20 mm/h), and immunoglobulin E levels were normal. Negative results were confirmed for infective four indexes, including hepatitis B, C, syphilis, and HIV.

Arterial blood gas analysis showed the pH to be 7.43 (NR: 7.35–7.45), partial pressure of oxygen to be 58.60 mmHg (NR: 80–100 mmHg), and partial pressure of carbon dioxide to be 37.40 mmHg (NR: 32–45 mmHg), whereas the lactate level was 2.78 mmol/L. Furthermore, serum tests for the detection of nine respiratory pathogens (Q fever rickettsial, influenza A virus, influenza B virus, respiratory syncytial virus, parainfluenza virus, *Mycoplasma pneumoniae*, *Chlamydia pneumoniae*, adenovirus, and *Legionella pneumophila*) yielded negative results.

Negative results were also obtained for the antinuclear antibody, anti-extractable nuclear antibody, antiphospholipid antibody, antineutrophil cytoplasmic antibody, rheumatoid factor, and anti-cyclic citrullinated peptide tests. Consistently, the sputum smear fungi, sputum smear bacteria, and acid-fast bacillus tests yielded negative results on three separate occasions, and negative results were also obtained for urinary protein, urinary erythrocyte, leukocyte, and stool occult blood tests, as well as for serum next-generation sequencing of bacteria.

**Figure 1:**
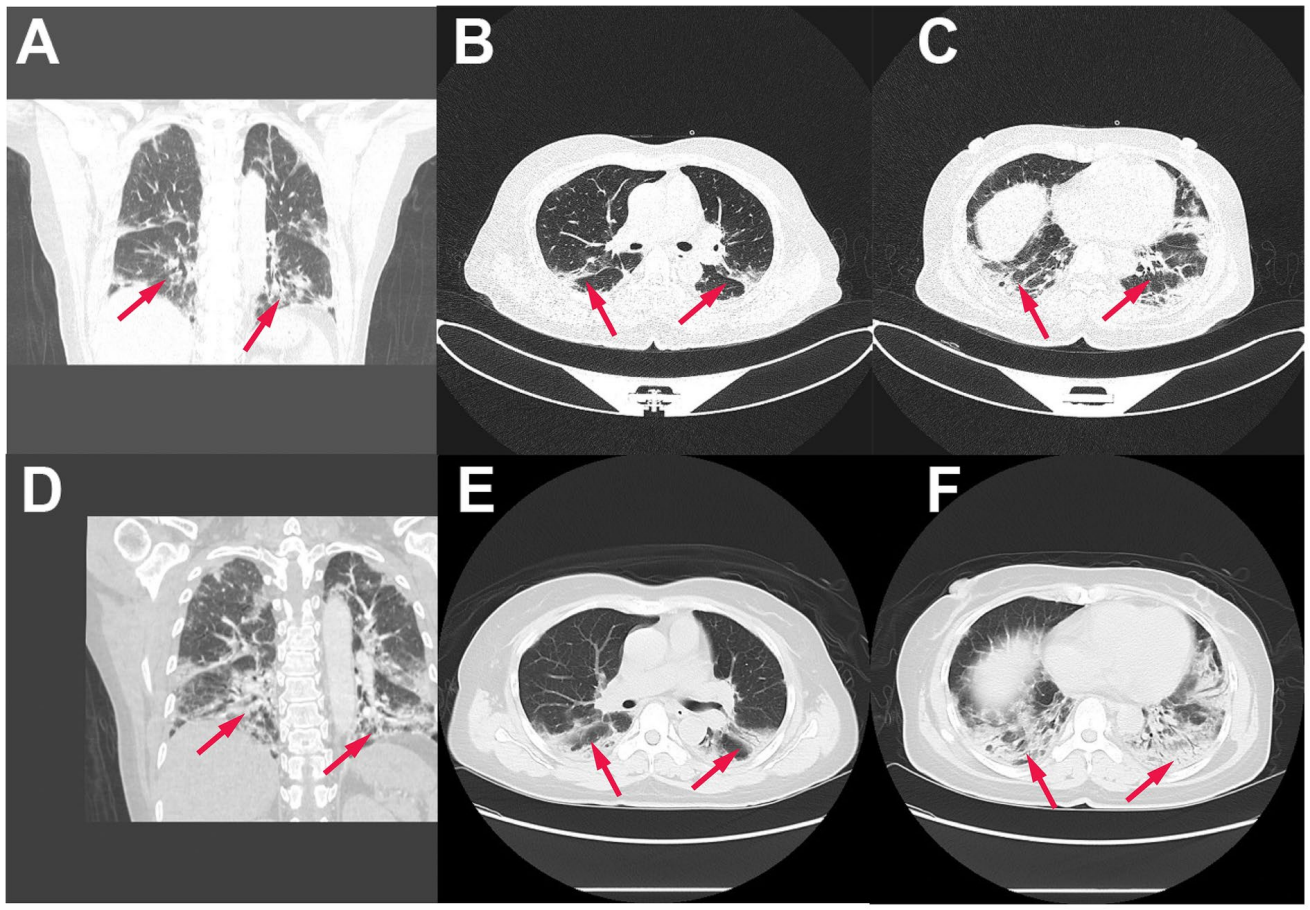
Pulmonary lesions at different intervals. **(A–C)** pulmonary computed tomography (CT) at admission shows diffuse bilateral patchy shadows and interstitial changes: coronal image **(A)** and axial slices from the lung apices to the bases **(B,C)**; **(D–F)**, pulmonary CT after 1 week reveals diffuse bilateral patchy shadows, ground glass opacities and interstitial changes: coronal image **(D)** and axial slices from the lung apices to the bases **(E,F)** (Red arrows).

Pulmonary function tests revealed a moderately restrictive pulmonary ventilation disorder, and computed tomography (CT) pulmonary examination performed on admission revealed multiple patchy shadows in the bilateral lung fields, partial bronchiectasis, and slight interstitial changes (Figures 1A–C). Although intravenous piperacillin-tazobactam [antibacterial: 4.5 g administered at 8 h intervals (q8h)], doxycycline, aerosolized budesonide, and levalbuterol were administered over the following 3 d, and the cough and wheezing were not alleviated. Subsequently, intravenous antifungal therapy with 300 mg of voriconazole and 40 mg of methylprednisolone was administered at 12-h intervals. Four days later, the patient’s shortness of breath gradually worsened, and despite oxygen at 8 L/min, she developed hypoxemia (peripheral oxygen saturation 90%). In addition, a repeat CT examination 1 week later after admission revealed further severe multiple patchy shadows, ground glass opacities, partial regions of confluent consolidations, and interstitial changes in the bilateral lung fields, with progressive lesion development (Figures 1D–F). Metagenomic sequencing of the bronchoalveolar lavage fluid sample revealed no pneumocystis carinii, viral RNA, viral DNA, bacteria, fungi, mycoplasmas, chlamydia, or parasites. Blood gas analysis indicated metabolic acidosis, and ventilator support was provided. The CT image findings indicated that autoimmune-featured ILD should be considered, particularly myositis-specific antibodies. Accordingly, serum was obtained from the patient for the detection of these antibodies. Profile analysis of myositis-specific autoantibodies (anti-Jo-1, anti-PL-7, anti-EJ, anti-OJ, anti-Zo, anti-KS, anti-Ha, anti-Mi-2α, anti-Mi-2β, anti-TIF1γ, anti-NXP2, anti-MDA5, anti-SAE1, anti-SAE2, anti-HMGCR, anti-SRP, anti-cN1A, anti-CENP-B, anti-Scl-70, anti-RNA-PIII, anti-Th/To, anti-NOR-90, anti-fibrillarin, anti-Ku, anti-PM-Scl100, anti-PM-Scl75, and anti-Ro-52) based on indirect immunofluorescence assays revealed the presence of anti-MDA5 antibodies (1:300; Figure 2). The patient was thus diagnosed with MDA5-positive DM and was accordingly intravenously administered methylprednisolone (total 500 mg/d) and oral tacrolimus 1 mg twice a day for 2 d in combination with cyclophosphamide (0.6 g). However, despite treatment, the patient remained hypoxic and dependent on a ventilator, and bedside chest radiography revealed scattered patchy shadows in both lungs, which was particularly pronounced in the lower left lung (Figure 3). However, for economic reasons, the patient and her family decided to discontinue treatment after a 2-week hospital stay.

**Figure 2:**
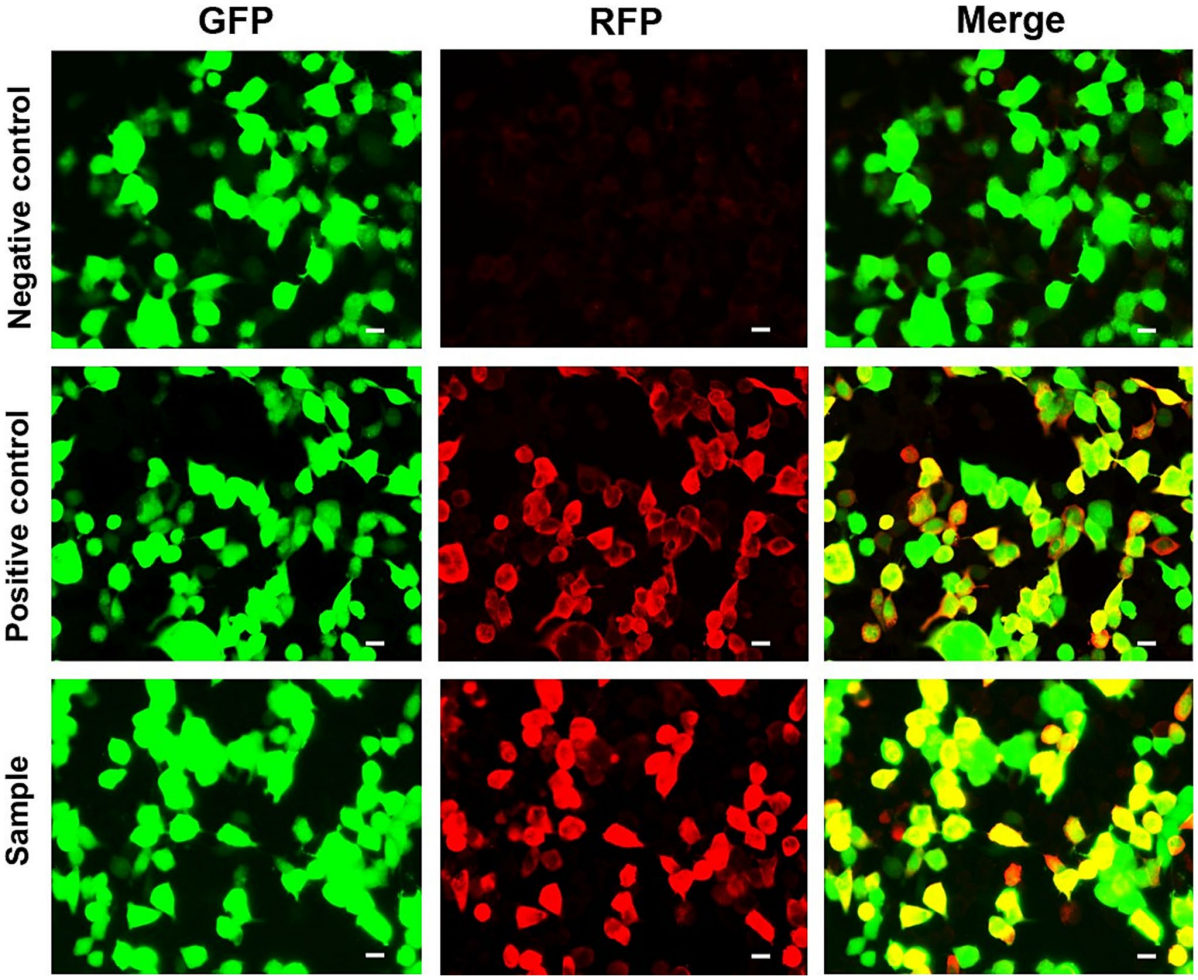
Indirect immunofluorescence detection of MDA5 antibodies. Green fluorescent protein (GFP) expression indicates the successful transfection of the plasmid. Red fluorescent protein (RFP) expression indicates anti-MDA5-positive antibodies. The patient’s blood sample was positive for GFP and RFP. Scale bar = 20 μm.

**Figure 3:**
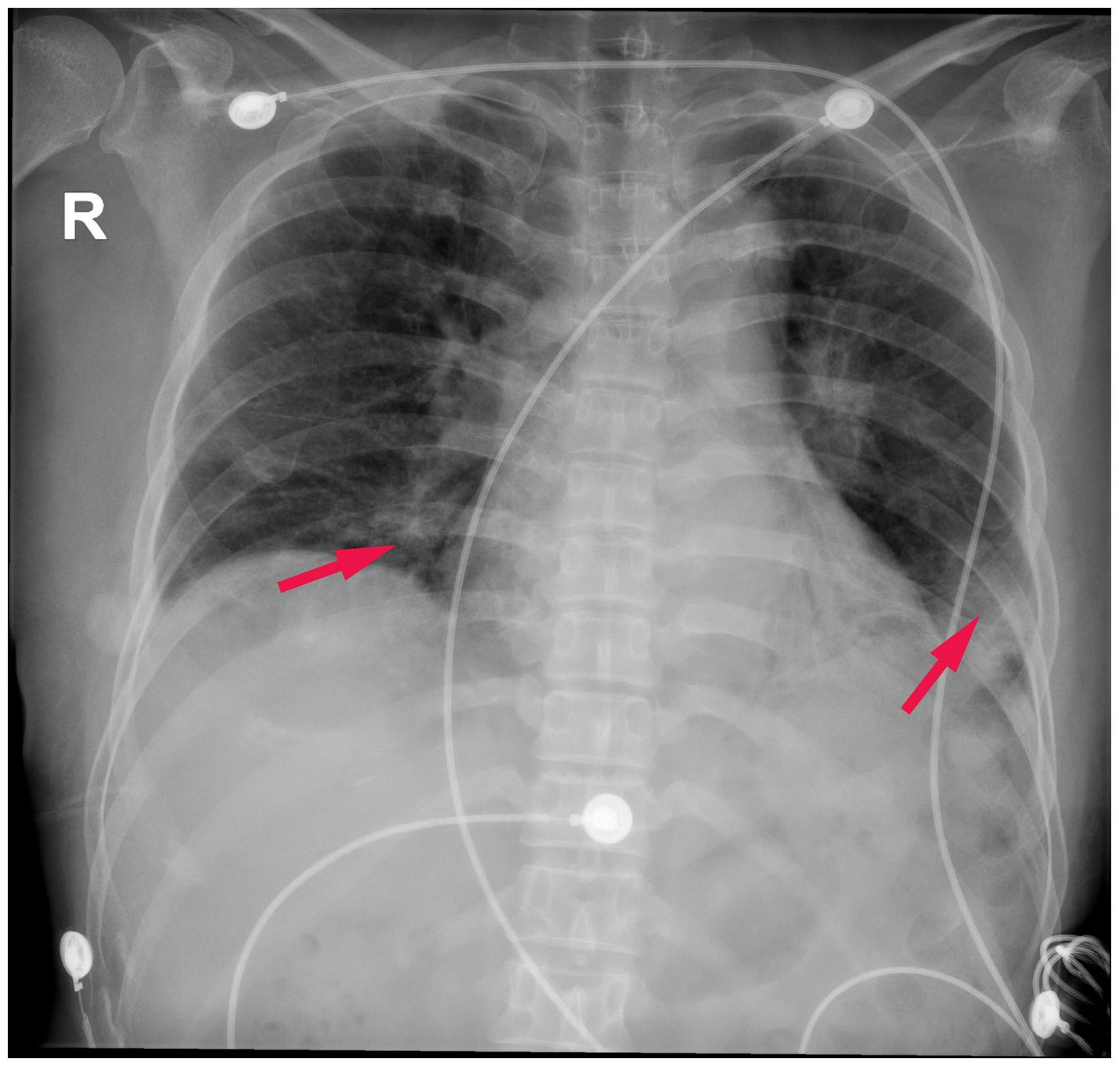
Posterior–anterior chest radiograph. Scattered patchy shadows in both lungs, which was particularly pronounced in the lower left lung (Red arrows).

## 3 Discussion

We report the case of an MDA5-positive patient, for whom the diagnosis was delayed owing to the absence of a typical rash and muscle weakness. After receiving a multidisciplinary consultation, the diagnosis of MDA5-positive DM was finally made. To gain further insights into rare cases such as this, we reviewed the cases of 11 anti-MDA5-positive patients with interstitial lung disease (ILD) without rash or myositis, which had been reported between 2016 and 2024 (Table 1). Of these patients, six were males and five females, nine of whom presented with respiratory symptoms, including dyspnea and cough, which were treated with glucocorticoid-based combination therapy. One patient was lost to follow-up, nine patients were died in the short-term, and only one patient surviving post-lung transplantation. In the absence of a specific standard treatment, most of these patients receive combined immunosuppressive therapy.

**Table 1:** Cases of MDA-5 positive dermatomyositis combined with interstitial lung disease but without myositis and rashes.

Author	Year	Sex, age (years)	Clinical manifestation	Therapy regimens	Prognosis and outcome
Asano et al. (26).	2024	Male, 93	acute dyspnea and dry cough	intravenous cyclophosphamide pulse of 500 mg oral tacrolimus 3 mg daily and high-dose gamma globulin therapy for 5 days	died on 2 months later
Mehta et al. (27).	2021	Female, 42	fever, cough, and shortness of breath for 1 month	rituximab 500 mg/day for 1 day on the 14^th^ day	died on the 23^rd^ day of hospitalization
Hong et al. (11).	2020	Female, 51	2-week history of dyspnea and polyarthralgia	prednisolone 37.5 mg/day and hydroxychloroquine 400 mg daily	discharged home, died from respiratory failure 1 week later
Pacot et al. (28).	2020	Male, 51	dyspnea	corticosteroids (500 mg) for 5 days, cyclophosphamide (600 mg/m^2^), plasmapheresis, lung-transplantation, mycophenolate, tacrolimus, methylprednisolone	alive at 3 years after lung transplantation
Aoyama et al. (29).	2019	Male, 47	cough and fever	methylprednisolone (1,000 mg/day) was administered for 3 days, IVCY (500 mg/body) and oral tacrolimus (5 mg/day)	died on hospitalization day 69
Sakamoto et al. (30).	2019	Female, 72/ Female, 68/ Male, 70	general fatigue/dyspnea and abnormal shadows on a chest radiograph/dyspnea	methylprednisolone pulse, cyclophosphamide/methylprednisolone pulse, cyclophosphamide/oral prednisolone and intravenous cyclophosphamide	died of respiratory failure 42 days after admission/died of respiratory failure 27 days after admission/died of respiratory failure 44 days after admission
Gonzalez-Moreno et al. (31).	2018	Female, 54	arthralgia and dyspnea for 2 weeks	methylprednisolone (five pulses of 500 mg/day) cyclophosphamide (1,000 mg)	improvement, discharged 1 month after admission.
Ortiz-Santamaria et al. (5).	2017	Male, 77	dyspnea	endotracheal intubation, corticosteroid	died
Chino et al. (32).	2016	Male, 56	fever and fatigue for 1 week	methyl-prednisolone pulse therapy	died

NA, not available.

The sex prevalence in anti-MDA5 DM varies among different ethnicities. It can occur in both males and females, with a slight female predominance (3). MDA5-positive DM represents less than 2% of idiopathic inflammatory myopathies in Europe and 11 to 60% in Asia (4). MDA5-positive DM is associated with several non-specific indicators, including those indicative of liver dysfunction, such as elevated alanine transaminase or gamma-glutamyl transferase, elevated ferritinemia, and a higher CD4^+^/CD8^+^ T ratio, all of which are correlated with the severity of the disease (5–8). Xie et al. (9) showed that old age, male sex, hypoxemia, low forced vital capacity, lymphocytopenia, and high levels of ferritin, CRP, creatine kinase, and LDH are risk factors for mortality in patients with MDA5-positive DM. In the case presented herein, serum glutamic oxaloacetic transaminase, glutamic pyruvic transaminase, *γ*-glutamyl transpeptidase, and LDH were elevated when assessed on admission.

MDA5 is activated by viruses and induces interferon (IFN) expression, thereby promoting increase in MDA5 levels and inducing inflammatory cytokines that propagate the immune response (10). Rapidly progressive ILD (RP-ILD) is commonly observed in patients with MDA5-positive DM, and such patients have worse baseline pulmonary function test scores than those with MDA5-negative DM. MDA5-positive idiopathic inflammatory myopathy is frequently complicated by rapid, progressive, and substantial mortality owing to respiratory failure (11), and CRP-to-albumin ratio, red blood cell distribution width-coefficient of variation, fever status, and CD3^+^ T cell counts have been reported to predict RP-ILD in patients with MDA5-positive DM (12). Niu et al. reported that duration, fever, pleural effusion, high total CT scores, and elevated aminotransferase collectively serve as reliable predictors of the prognosis of MDA5-positive DM-ILD (13), whereas Li et al. reported that the serum level of sCD206 is more appropriate for evaluating progression than ferritin levels in patients with MDA5-positive DM complicated by ILD (14). Furthermore, Liu et al. showed that IFN-beta and eukaryotic translation initiation factor 2-alpha kinase 2 can be used as potential therapeutic targets, because of their role in the pathogenesis of MDA5-positive DM-ILD (15). So et al. showed that a serum LDH level  >300 IU/L is an independent risk factor for RP-ILD, and high LDH levels may reflect ILD severity in patients that were MDA5-positive DM (3). However, the findings of an investigation examining the clinical features and long-term prognosis of a large, single-center adult MDA5-positive DM North American cohort failed to indicate any demographic, serological, or clinical features associated with long-term remission (16). In the case of the patient reported herein, there was no evidence of a rash typically associated with this disease and she presented with non-specific symptoms such as cough before admission and fever after admission. Notably, however, a rapid progression of ILD was apparent within a short duration after admission. Previous studies have reported that current EULAR/ACR 2017 IMM criteria may miss the diagnosis of anti-MDA5 DM (17). We suggest that separate classification criteria are needed for anti-MDA5 DM, and all patients with RP-ILD should be tested for anti-MDA5 antibody.

Numerous studies have been conducted with respect to the treatment of DM (18–20). A multicenter, prospective, open-label, historical-controlled randomized study in Japan showed that the initial triple therapy strategy of high-dose glucocorticoids combined with intravenous cyclophosphamide and calcineurin inhibitors was effective in subsequent retrospective studies, and could improve patients’ lung function (21). Our patient received high-dose glucocorticoids combined with intravenous cyclophosphamide and tacrolimus triple therapy. However, the patient abandoned the treatment due to economic reasons and was discharged. Early initiation of plasma exchange combined with intensive immunosuppressives has been found to be effective for patients with DM and refractory RP-ILD (22). Furthermore, Holzer et al. reported a case in which the use of daratumumab significantly alleviated pulmonary lesions in severe antibody-mediated autoimmune diseases, such as MDA5-positive DM (23), whereas Jiang et al. describe an MDA5-positive patient who was treated with tofacitinib during maintenance therapy (24), and Phillips et al. showed that co-trimoxazole reduces mortality in patients that are MDA5-positive DM (25). The treatment of the patient described herein included the administration of intravenous methylprednisolone and cyclophosphamide, although the ultimate efficacy could not be ascertained, given that the patient refused to undergo treatment beyond the initial 2 weeks. New and effective treatments for MDA5-positive DM and associated disorders are urgently needed to reduce mortality rates and improve the quality of life of the patient.

## 4 Conclusion

Atypical MDA5-positive-DM-ILD in the absence of a rash and muscle weakness needs to be diagnosed and reported more accurately. A timely diagnosis followed by appropriate treatment is key to reducing the high rates of mortality associated with this disorder.

## Data Availability

The original contributions presented in the study are included in the article/supplementary material, further inquiries can be directed to the corresponding author.
